# A critical review of chronic kidney disease as a risk factor for coronary artery disease

**DOI:** 10.1016/j.ijcha.2021.100822

**Published:** 2021-06-15

**Authors:** Mark Coyle, Gerard Flaherty, Catriona Jennings

**Affiliations:** School of Medicine, National University of Ireland Galway, Galway, Ireland; National Institute for Prevention and Cardiovascular Health, Galway, Ireland

**Keywords:** Coronary artery disease and chronic kidney disease, Cardiorenal syndrome, Cardiovascular prevention, Coronary artery disease progression

## Abstract

Chronic kidney disease (CKD) is a significant risk factor for cardiovascular disease (CVD). In addition to common CVD risk factors, the presence of CKD is independently associated with an elevated cardiovascular (CV) risk. We examined the association between CKD and CVD, focusing on coronary artery disease (CAD) in both primary and secondary CVD. A total of 94 articles were included for this review using search strategies on Pubmed and Google scholar. The main findings of our review included that besides sharing common risk factors, CKD induces several physiological microscopic changes leading to increased CV risk. These microscopic changes manifest macroscopically with evidence of the development of primary CAD in CKD patients, in addition to accelerating CAD in those with pre-established CV pathology, with CKD consequently being a risk factor for both primary and secondary CAD progression. Current CV guideline recommendations do not discriminate between those patients with and without CKD. Future research is needed in this area, examining if there may be a role for tighter modifiable risk factor targets in this high-risk population.

## 1 Main

CVD is the single most common cause of death globally and it accounts for almost half of the 36 million non-communicable disease deaths per year [1]. Chronic kidney disease (CKD) is defined by Kidney Disease: Improving Global Outcomes (KDIGO) as an abnormality in kidney structure or function present for more than three months, with implications for health [2]. CKD is a global health burden with a worldwide prevalence of approximately 13.4% [3]. CKD has many implications for health, particularly CV implications. Patients with CKD are more likely to die from CVD than to progress to end-stage renal disease (ESRD) [4] and CVD is the leading cause of mortality and morbidity in CKD patients [5]. Both CAD and CKD have shared risk factors [6], however, the presence of these risk factors alone does not explain the inflated risk of CVD. CKD is itself an independent risk factor for CAD. This literature review will analyse the relationship between CKD and CAD, discuss evidence for CKD in both primary and secondary CAD prevention, and highlight gaps in the current literature for this important topic.

### 1.1 Search strategy

A literature search was conducted using Pubmed and Google Scholar electronic databases. Search terms included “coronary artery disease and chronic kidney disease,” “cardiovascular disease and chronic kidney disease,” “cardiorenal syndrome,” “relationship,” “primary prevention,” “secondary prevention,” “modifiable risk factor targets,” “management” and these terms were used in different combinations. Articles were reviewed and assessed for relevance. Articles from database inception to 2020 were included. Our final search strategy included all published papers and those with the highest quality of evidence such as *meta*-analyses and randomised controlled trials (RCT) were given priority to be selected for inclusion. The inclusion criteria also included articles with accessible abstracts and full texts.

### 1.2 Findings

#### 1.2.1 Pathophysiology of CVD in patients with CKD

Besides shared classic CVD risk factors such as hypertension, diabetes and advancing age, CKD causes several physiological changes that are associated with the excess risk of CVD in this population. The relationship between CKD and CAD is complex and not fully understood, but these changes include “non-traditional” risk factors, uraemic toxins, vascular calcification and arterial remodelling. A review paper published in 2014 detailed the “non-traditional” risk factors associated with CKD which consist of anaemia, oxidative stress and inflammation [6]. The role of anaemia is significant, as anaemia is an independent risk factor for left ventricular hypertrophy (LVH) [7]. Despite this, management of anaemia in CKD is complex. The CREATE trial showed that complete normalisation of haemoglobin did not have any additional cardiovascular benefit compared to the lower target range of 10.5 to 11.5 g per deciliter [8]. Furthemore, the TREAT trial showed that use of darbepoetin alfa in patients with type 2 diabetes and CKD in order to increase haemoglobin levels resulted in no reduction in adverse CV events [9]. Part of the remaining risk in “cardiorenal syndrome” is hypothesised to be due to uraemic toxins [10] which have been shown to cause inflammatory changes, enhanced collagen synthesis and endothelial dysfunction [11], [12], [13]. These protein-bound uraemic toxins cause renal inflammation and fibrosis, enhanced cardiac collagen and protein synthesis, ultimately leading to endothelial dysfunction [6]. Histological examination of coronary artery plaques of patients with end stage renal disease (ESRD) at autopsy have shown atherosclerotic lesions in ESRD to be uniquely more calcified than fibroatheromatous, with significantly reduced lumen areas and increased plaque calcification compared to non-uraemic subjects [14]. Additionally, the plaques had a greater media thickness in comparison to lesions in those without CKD. This is contrary to previous belief that size was the major difference in these lesions, as opposed to plaque composition [14]. This plaque calcification is postulated to be attributed to alterations in mineral-bone metabolism, commonly seen in CKD and referred to as CKD-mineral bone disorder (CKD-MBD), which leads to another phenomenon increasing the risk of CVD – arterial calcification [15]. Results of a *meta*-analysis have shown such vascular calcification to be associated with an increased risk of CVD [16]. This inappropriate calcification causes stiffening of the arterial wall, raised pulse pressure, and LVH [16] and is enhanced by metabolic insults caused by CKD, leading to “osteoblast-like” cells forming in the vessel wall [17]. Finally, patients with CKD also have a high prevalence of arteriosclerosis and remodelling of large arteries. A major CV research interest in CKD has been comparing pathology leading to arteriosclerosis in CKD - medial calcification and a relative lack of inflammatory cells - to “conventional” atherosclerosis in those without CKD. The “non-traditional” risk factors culminate in thickening and calcification of the vessel wall and these noncompliant vessels lead to elevated systolic blood pressure (SBP), raised pulse pressure, LVH, and reduced coronary blood flow [18]. Epidemiological studies have demonstrated these consequences to be predictors of major adverse cardiac events (MACE) in ESRD patients [19]. These microscopic changes are summarised in Table 1. Overall it is a complex relationship, but undoubtedly there is more to the increased risk in CKD than simply shared risk factors.

**Table 1:** Proposed mechanisms of atherosclerosis/cardiovascular manifestations in patients with chronic kidney disease.

Microvascular changes in CKD	Cardiovascular manifestation
Uraemia, uraemic toxins	Enhanced collagen synthesis, vascular calcification, endothelial dysfunction
Mineral bone disorder, hyperphosphataemia	Arterial calcification, LVH, diastolic dysfunction
Macrophage activation	Accelerated atherosclerosis
Activation of the RAAS	Hypertension, LVH
Malnutrition, anaemia, hyperhomocysteinemia, elevated fibrinogen	Atherogenesis

CKD, chronic kidney disease; LVH, left ventricular hypertrophy; RAAS, renin angiotensin aldosterone system.

#### 1.2.2 CKD as a risk factor in primary CAD prevention

Evidently at a microscopic level, the pathophysiological changes induced in CKD lead to an increased risk of CAD. This has been proven on a macroscopic level, with levels of kidney function being shown to be associated with CV risk. A longitudinal study of subjects with a diverse range of eGFR showed the level of eGFR to be an independent risk factor for CVD [20]. These findings are consistent with another longitudinal United States (US) study which showed an inverse relationship between reducing eGFR and risk of MACE [21]. Milder impairments in renal function have also been shown to increase the risk of CAD with higher levels of eGFR being linked to a reduction in onset of CVD across all ages [22]. Similarly, in female patients presenting with chest pain, renal insufficiency was a predictor of significant coronary disease on angiography [23]. The Systolic Blood Pressure Intervention Trial (SPRINT) demonstrated that CKD is an independent risk factor for CVD, showing over a three year follow-up participants with CKD had a higher number of adverse CV events [24]. However, the relationship between kidney function and outcomes has been more ambiguous in some low-risk patients. In the Framingham Study and the first National Health and Nutrition Examination Survey (NHANES I) mild renal dysfunction was not shown to be an independent risk factor for CVD [25], [26]. Contrary to this, renal dysfunction was found to be an independent risk factor for CVD in the Atherosclerosis Risk in Communities Study [27] and NHANES II [28]. These differences could possibly be related to dissimilar study populations and type II errors owing to reduced numbers of MACE rates in community studies, as has been previously described [29]. Nonetheless, the overwhelming evidence is in favour of CKD being an independent risk factor for CVD [30].

#### 1.2.3 CKD as a risk factor in secondary CAD prevention

Clearly, CKD is a risk factor for developing CAD. The significance of CKD in those with established CVD will now be discussed. CKD impacts outcomes after ACS in the immediate, intermediate and longer term.

In the early stages after ACS, there is evidence that CKD elevates CV risk. Analysis of a database of four large RCTs showed that at both 30 days and six month follow-up, renal dysfunction independently predicts MACE [31]. In a Japanese ACS cohort, patients with CKD had a much higher in-hospital cardiac mortality rate than those without CKD, and in the presence of anaemia, a feature of CKD, they had an even higher mortality rate [32]. This elevated in-hospital mortality risk has been shown in retrospective and prospective studies with mortality ranges of 2% in those with normal renal function to 30% in those with ESRD [33], [34]. Similar findings were demonstrated in patients with stable CAD over six months, showing a potent relationship between the level of CKD and death from CVD [35]. Interestingly, investigation of non-culprit lesions following PCI, using intravascular ultrasound over eight months indicated that CKD stages 3–5 were independent predictors of plaque advancement while plaque regression occurred in stages 1–2 [36]. Similarly, an observational study in the US of patients with MI showed that moderate CKD increased the risk of mortality for the first six months however, there was no difference in risk after this [37]. Analysis of the database of two RCTs identified baseline renal dysfunction as a parameter to be incorporated in a risk score, which accurately predicts 30-day and one-year mortality after MI [38]. Contrary to these findings, another RCT trial showed no significant difference in mortality at nine months in those with mild-moderate renal impairment after percutaneous coronary intervention [39]. Furthermore, ESRD has been shown to have double the rate of restenosis after coronary stenting than a control group [40].

In the intermediate term there is also evidence that CKD offers a poor prognosis in secondary CVD prevention. Analysis of ACS patients at one year follow-up showed there were 3.5% more ischaemic events in those with CKD [41]. This has also been demonstrated in the Taiwan population and in other studies where one year and beyond after MI, CKD was independently linked to MACE as well as all-cause mortality [42], [43], [44], [45]. Analysis of the Israeli ACS population indicated that poorer renal function was independently associated with an excess mortality risk of between 25% and 40% for each decrement of 10 U of eGFR at one year [46].

The impact of renal dysfunction has been shown to have a negative effect on patients with ACS in the longer-term, too. Severe CKD was an independent predictor of MACE when patients were followed-up for 500 days [47]. Analysis of a RCT looked at the relationship between reduced renal function and CV outcomes post-MI over two years and showed a considerably elevated cardiac risk in those with CKD [48]. An Irish community-based study of patients with CVD linked a 10 mL/min reduction in eGFR in CKD to a 20% increase in MACE when patients were followed for almost three years [49]. In another RCT, the risks of ischaemic events increased markedly with declining CKD stages over 30 months following ACS, shown again in a study over three years [50], [51]. Analysis of data from four large longitudinal studies demonstrated that CKD in community-based populations with CVD is associated with an increased risk for recurrent MACE over 86 months [52]. Even early-stage CKD was a major risk factor for long-term CV death after ACS when patients were followed up for just over four years [53] and continuous deterioration of kidney function over five years was linked to an increased risk of CV mortality [54], [55]. The study of a Danish registry over six years found that mortality in patients post-ACS only increased when creatinine clearance was<40 mL/min [56]. Findings from four major community-based studies over ten years concluded that a combination of CKD and CVD was very high-risk for MACE [57].

Despite being identified as a high-risk population post-ACS, CKD patients remain relatively undertreated [58]. An American study showed that in CKD there was a lower use of secondary CVD preventive medications, with ESRD patients having the lowest use, both within the first 24 h and at discharge [33], [59]. This is similar to previous US studies, with less uptake of evidence-based medical therapy in those with CKD post-ACS [60]. CKD predisposes patients to bleeding and higher levels of in-hospital bleeding have been exhibited previously, a possible explanation for the less aggressive strategy sometimes adopted [60]. An observational study of the SWEDEHEART registry to determine the safety of dual antiplatelet therapy (DAPT) in CKD patients in the short versus long-term was carried out. Those who continued the DAPT in the longer-term had a reduced risk of MACE but an elevated risk of bleeding. However, overall the study favoured a longer-term DAPT approach [61]. As well as showing a lower prescription rate of drugs, analysis of the Swedish coronary population suggested that CKD patients were more likely to discontinue treatment [62]. Patients with ESRD receive less aggressive treatment than patients with normal renal function [33] and their excess mortality could be attributed, partly, to the lower rate of use of these drugs [63]. In the Israeli ACS population, as the eGFR reduced patients were less often treated with these secondary preventive medicines despite having higher mortality rates [64].

However, this is an over-simplified argument for an intricately complex subject. Haemostasis and functioning of the coagulation system are profoundly altered in CKD [65]. These patients are prone to both bleeding and excessive thrombosis [66]. Dysfunctional coagulation results from altered platelet function, modification of the coagulation cascade and stimulation of fibrinolysis, while hypercoagulability is produced by disordered regulatory factors and increased platelet reactivity [65]. In patients with ACS, impaired renal function has been shown to be associated with increased risks of bleeding [67] and aspirin induced prolonged bleeding times [68].

An RCT indicated that intensive management of ACS in patients with CKD/ESRD may be favourable and suggested that clopidogrel was safe and beneficial with and without CKD [69] but more studies are needed in this area [70]. A *meta*-analysis concluded that post-ACS, DAPT reduced MI but there is uncertainty when balanced against the risk of bleeding in CKD [71]. CKD patients, particularly those with ESRD, have been excluded from the majority of CHD trials [72], so the question whether a more aggressive approach in managing this high-risk population can improve outcomes should be further investigated.

### 1.3 Modifiable risk factor targets in CKD

Evidently, the CKD population have a very high CV risk. Therefore, would tighter targets be desirable in this group of patients? This review will now focus on current evidence and guidelines for specific modifiable risk factors for CAD in CKD: hypertension, hyperlipidaemia, diabetes and CKD-MBD.

#### 1.3.1 Hypertension

The SPRINT Trial showed that a SBP target of 120 mmHg resulted in significant reductions in MACE, including in patients with CKD, when compared to the traditional target of 140 mmHg [73]. However, there was a higher incidence of adverse kidney events in the more aggressively treated group. Only a third included had CKD, so similar investigations specifically for CKD would be mandatory if existing guidelines for CKD are to be challenged [74]. Similarly, a *meta*-analysis showed a positive correlation between reduction in SBP and CV risk, even in those with lower baseline SBP (<130 mmHg) [75]. Contrary to this, a RCT showed that BP-lowering treatment did not reduce the risk of MACE in patients with baseline SBP values in the high–normal range [76]. Another RCT indicated that reducing SBP to <120 mmHg was actually associated with increased numbers of MACE [77]. Critically, in the SPRINT trial and *meta*-analyses that have shown reduced CV risk by diminishing ‘baseline’ BP in the high–normal range, ‘baseline’ was frequently calculated on a background of antihypertensive medication. Therefore, these studies do not provide evidence to support treatment initiation in patients without hypertension. Current ESC guidelines for hypertension recommend primarily lowering BP to <140/90 mmHg and if well-tolerated lower, but not <120 mmHg [78]. Current American Heart Association (AHA) guidelines recommend a blood pressure target of <130/80 mmHg for patients with CKD [79]. The KDIGO blood pressure guidelines published in 2021 recommend a target SBP of <120 mmHg, when tolerated, in those with hypertension and CKD [80].

#### 1.3.2 Lipids

The ESC 2019 guidelines described new, more aggressive targets for low density lipoprotein cholesterol (LDL-C). In those at very high CV risk, a reduction of more than 50% with a target of <1.4 mmol/L is desirable. An even-lower target of <1 mmol/L is recommended for patients experiencing a second vascular event within two years, while taking a maximum tolerated dose of a statin [81]. AHA guidelines are based on overall CV risk, influenced by the presence of other risk factors in addition to CKD. Those in the very high risk category are recommended to reduce LDL cholesterol by greater than 50% while for those in the intermediate risk category, a reduction of 30–49% is desirable [82]. A *meta*-analysis looking at the safety and efficacy of more intensive LDL-C lowering with statins concluded that for every 1 mmol/l reduction there was a reduction in annual MACE by over a fifth, and no threshold was identified [83]. Targeting a lower-than-recommended level has been proposed to be additionally beneficial with no negative effect by means of statin add-on therapies [84]. This was shown in RCT with ezetimibe [85] and alirocumab [86]. A RCT showed evolocumab use on a background of statins reduced MACE when LDL-C was decreased to levels of 0.78 mmol/L, connoting benefit for even lower levels than current recommendations [87]. The SHARP trial demonstrated significant benefit for lipid lowering therapy as primary CV prevention in non-dialysis patients, with the use of simvastatin plus ezetimibe safely reducing the incidence of MACE in patients with advanced CKD [88]. The KDIGO 2013 guidelines for lipid management in CKD recommend assessment of 10-year CV risk of all patients not on a statin and commencement of a statin if necessary based on their level of overall CV risk. A full lipid profile is obtained at the initiation of treatment but follow-up lipid levels are not necessary, and specific LDL-C targets are not recommended [89].

#### 1.3.3 Glycaemic control

It has been shown in a *meta*-analysis that intensive glycaemic control results in a considerable reduction in MACE [90]. Current European and American diabetes guidelines recommend an overall target for HbA1c of <7%, but that targets should be individualised [82], [87]. A lower target should be considered, particularly in the young at no risk of hypoglycaemia, while a more relaxed target of <8% may be appropriate in the elderly [91]. An RCT showed there was actually an increase in mortality when HbA1c levels of <6% were targeted, and there was no reduction in death from CVD [92]. In contrast, the KDIGO 2020 guidelines for diabetes management in CKD recommended and individual HbA1c target ranging from <6.5% to <8% depending on individual characterisitics [93].

#### 1.3.4 CKD-MBD

The 2017 KDIGO guidelines for CKD-MBD recommended use of lateral abdominal radiograph and echocardiography to investigate for vascular and valvular calcification respectively [94]. Treatment is targeted at lowering elevated serum phosphate and maintaining normal serum calcium levels, through dietary phosphate restriction and phosphate binders. Additionally, calcimimetic, calcitriol and vitamin D analogue usage is recommended for those needing parathyroid hormone lowering therapy. [94] The EVOLVE trial examined the effect of cinacalcet use on reducing CVD in CKD patients undergoing dialysis. Overall, the trial was nondefinitive, the unadjusted intention-to-treat analysis showed a non-statistically significant reduction in CV events, however the adjusted analyses showed that this reduction was nominal [95]. Table 2 summarises and compares current European, American and KDIGO guidelines for blood pressure, lipids and glycaemic control.

**Table 2:** Clinical guidelines for CVD risk factor modification in CKD.

Risk Factor	ESC	AHA	KDIGO
Blood Pressure	<140/90 mmHg, if well tolerated < 130/80 mmHg	<130/80 mmHg	Systolic < 120 mmHg
Lipids	eGFR < 30 mL/min/1.73 m^2^LDL < 1.4 mmol/L eGFR 30–59 mL/min/1.73 m^2^ LDL-C < 1.8 mmol/L	Very high risk: LDL-C reduction > 50% Intermediate risk: LDL reduction 30–49%	No LDL-C target
Glycaemic control (HbA1c)	<7% (<53 mmol/mol)	<7% (<53 mmol/mol)	<6.5% to < 8% depending on individual factors

CVD, cardiovascular disease; CKD, chronic kidney disease; ESC, European Society of Cardiology; AHA, American Heart Association; KDIGO, Kidney Disease Improving Global Outcomes; eGFR, estimated glomerular filtration rate; LDL-C, low density lipoprotein cholesterol.

This hypothesis of intensive management has been tested in a study before; CKD patients were assigned to two groups - aggressive risk factor modification and standard care. A dobutamine stress echocardiogram was performed on participants at baseline and at follow-up for evidence of new ischaemia or cardiac events over almost two years. The development of new ischaemia was common, but there was no difference between the standard and intensively treated groups [96]. Additionally, the recently published ISCHEMIA trial comparing initial invasive versus initial conservative strategies in those with stable coronary disease, advanced CKD and moderate or severe ischaemia demonstrated that there was no reduction in CV death or non-fatal MI between groups [97].

### 1.4 Summary of main findings

**Fig. 1:**
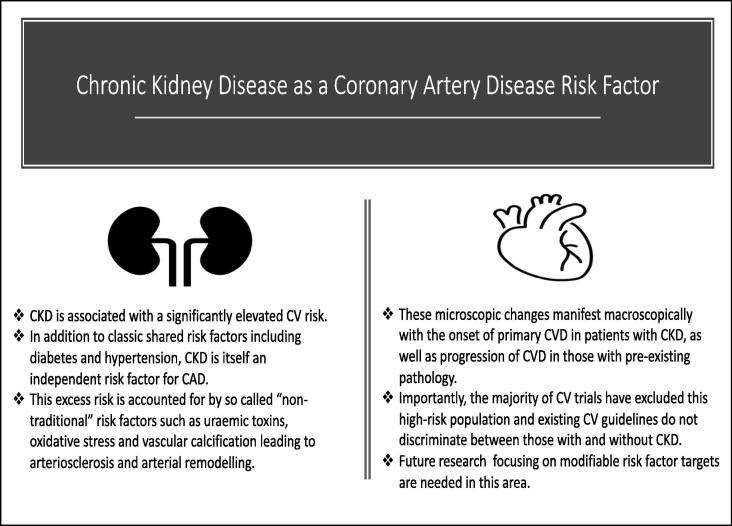
Chronic kidney disease both shares risk factors and acts independently as a risk factor for coronary artery disease.

Approximately one seventh of the world population has CKD and the most frequent cause of death in these patients is CVD. CKD shares common “well-known” modifiable and non-modifiable risk factors with CVD. Additionally, CKD itself enhances CV risk, both in primary and secondary CAD. The pathophysiology of CAD development and progression in CKD is complex and not fully understood, but includes additional “non-traditional” as well as traditional risk factor and their interactions lead to a synergistic detrimental effect. Ultimately, those patients with established CAD and CKD are at very high-risk of future ischaemic CV events. Correspondingly, management of modifiable risk factors is imperative to prevent coronary disease progression and recurrence. A combined pharmacological and non-pharmacological approach is optimal for prevention of CV events. There is a discrepancy between current KDIGO and European and American CV guidelines in relation to specific modifiable risk factor targets in this population. Our findings are summarised in Fig. 1

### 1.5 Implications for future research or clinical practice

Evidently, CKD patients are a high-risk cohort of patients. Identification of these patients, promoting healthy lifestyle habits, aggressive risk factor modification and guideline-based treatment is essential in this group. Given that this group of patients would derive benefit from individualised risk factor modification, consideration to include these patients in structured prevention and rehabilitation programmes may have a role in their overall management. Significantly, the high-risk nature of this population has meant their exclusion from the majority of CV trials. Consequently, future research including this population is needed. Challenging modifiable risk factor targets in this population may be a research area of interest, to determine if lower than currently advised targets would derive more CV benefit in this high-risk group, to ascertain if coronary disease progression can be impeded.

## 2 Conclusion

CKD is an important and independent risk factor in both primary and secondary CVD prevention. CKD results in an accelerated CAD course and it is important that physicians treating patients are aware of this enhanced risk and treat risk factors aggressively to reduce disease progression and recurrence. Future research and clinical trials are necessary in this population, focusing on risk factor targets, with possible implications for future guidelines.

## Funding

This research did not receive any specific grant from funding agencies in the public, commercial, or not-for-profit sectors.

## Declaration of Competing Interest

The authors declare that they have no known competing financial interests or personal relationships that could have appeared to influence the work reported in this paper.
